# Efficacy of Intra-articular Hyaluronic Acid and Triamcinolone Acetonide Combination Versus Hyaluronic Acid Alone in Knee Osteoarthritis

**DOI:** 10.7759/cureus.96129

**Published:** 2025-11-05

**Authors:** Omorogiuwa I Oduware, Alfred O Ogbemudia, John E Onuminya

**Affiliations:** 1 Department of Orthopaedics and Traumatology, University of Benin Teaching Hospital, Benin City, NGA; 2 Department of Orthopaedics and Traumatology, University of Benin, Benin City, NGA; 3 Department of Orthopaedics and Trauma, Irrua Specialist Teaching Hospital, Irrua, NGA

**Keywords:** hyaluronic acid, injection treatment, knee osteoarthritis, outcomes, triamcinolone

## Abstract

Background: Osteoarthritis (OA) is a chronic debilitating joint pathology and a major cause of disability worldwide. Treatment of knee OA primarily focuses on pain control and improvement of the quality of life of the patient. With the continuous increase in elderly population worldwide and consequent rise in knee OA, there is the need for new treatment strategies and optimal combination therapies for the treatment of patients suffering from early knee OA. Intra-articular injection of hyaluronic acid and corticosteroids has been demonstrated to be individually efficacious in the treatment of knee OA; however, they are both limited in the extent and duration of symptom relief.

Objectives: This study compared the efficacy of combined intra-articular injections of hyaluronic acid and triamcinolone versus hyaluronic acid alone in pain relief of Kellgren and Lawrence (K & L) grade 2 and 3 knee OA.

Methods: This was a hospital based randomized controlled double-blind study which included patients in attendance at the orthopaedic clinic, University of Benin Teaching Hospital (UBTH) with the diagnosis of Kellgren and Lawrence grade 2 and 3 Knee OA. The total sample size was 66, divided into two groups (A and B) of 33 respondents each. Patients in group A received combined hyaluronic acid, triamcinolone, lidocaine and sterile water intra-articularly into the affected knee, while those in group B received hyaluronic acid, lidocaine and sterile water only. Patients in both groups were evaluated using the 11 point numerical rating pain scale(NRS) and the short-form Western Ontario and McMaster Universities Osteoarthritis Index (WOMAC) function scale for pain and physical function status respectively at 0 week (prior to the injections) and 2, 4, 12 weeks after administration of the various agents. These were analyzed using the SPSS software, version 22 (IBM Corp., Armonk, NY). A p-value of <0.05 was considered to be statistically significant.

Results: Patients in both groups had progressive improvement in their pain scores and physical function following intra-articular injections. However, the pain and physical function scores decreased more rapidly and to a much lower levels in the group A patients who received the combined treatment than in the group B patients who received hyaluronic acid alone (p=0.001).

Conclusions: The results from this study suggest a synergistic effect between hyaluronic acid and triamcinolone, thus making them a more efficacious treatment option compared to hyaluronic acid alone in the treatment of K & L grade 2 and 3 knee OA. The findings from this study were based on a 12-week follow-up, more studies are encouraged to validate the findings over a longer period.

## Introduction

Osteoarthritis (OA) is a chronic, debilitating joint pathology characterised by gradual loss of articular cartilage [1]. Knee OA is one of the most common chronic synovial joint disorders that adversely affects the quality of life of the patient [2]. It is a major cause of disability worldwide with increasing prevalence during midlife [3-5]. The number of people with symptomatic knee OA is projected to be one in two at age 85 years [4]. Treatment of knee OA focuses mainly on pain reduction and improvement of the quality of life of the patient [6,7].

Hyaluronic acid (HA) is a high molecular weight substance found endogenously in the cartilage and synovial fluid of joints [7,8]. Its main functions include lubrication, scavenging of free radicals, and protein binding [9]. As OA progresses, endogenous HA depolymerises, decreasing its concentration and molecular weight, resulting in a reduction in the mechanical and viscoelastic properties of the synovial fluid in the affected joint [7-9]. Intra-articular hyaluronic acid helps in modifying the structure of the cartilage in knee OA, hence it is approved for the treatment of mild to moderate knee OA following NSAID failure [10,11]. Intra-articular corticosteroid injections have been found to reduce OA pain by inhibiting inflammation and reducing prostaglandin synthesis; thus, they are also recommended by several clinical practice guidelines as a safe and effective non-surgical treatment option for knee OA [12-15]. They have a very fast onset of action; however, their effect is short-term [15].

Topical or oral non-steroidal anti-inflammatory drugs (NSAIDS), paracetamol, and opioids are first-line pharmacotherapy in the treatment of knee osteoarthritis. While NSAIDS are associated with adverse side effects such as gastrointestinal perforations and cardiovascular events, opioid use can result in dependence, drowsiness, and mortality, which leads to a poor compliance rate [16-23]. Thus, this study seeks to find out if synergistic beneficial effects exist when intra-articular hyaluronic acid and steroids are combined as compared to intra-articular hyaluronic acid use only. Several studies have enumerated the benefits of intra-articular hyaluronic acid, with some comparing the benefits with intra-articular injection of steroids; however, only a few have studied the combined effects of both in the treatment of early knee OA, with their findings inconsistent [24-29]. Thus, this study was to help evaluate if both drugs can complement each other, resulting in early and sustained clinical response when used in the treatment of knee OA.

In a randomised clinical trial by de Campos et al. among 104 patients who were randomized into two groups of triamcinolone plus HA, and HA only, it was discovered that the addition of triamcinolone to HA improves first week symptom of pain and physical function compared to HA only, however the improvements were noticed to be the same beyond the first week for both groups [26]. Furthermore Ozturk et al. in a one year single-blind, randomised study done in Turkey to determine the safety and efficacy of intra-articular HA with or without corticosteroids in knee OA, found that both patient groups of HA plus triamcinolone and HA only group had improvement for both pain and physical function, however the HA plus triamcinolone group had superior early pain relief compared to the HA alone group (knee pain significantly improved at the second month) [27]. Also, MRI findings revealed that both treatments were safe, as no progression of cartilage damage was observed. However, there was discordance in the subgroup sample size as the sample size was in a ratio of 3:2 in favour of the combined group, which may have influenced the outcome of the study.

Similarly, Petrella et al. in a prospective, multicentre, randomised cluster bond feasibility trial found that combined HA and corticosteroid provides better and rapid pain relief compared to HA alone [28]. The improvement in pain was observed in the combined HA plus corticosteroid group as early as two weeks, compared to six weeks in the HA group alone. However, both were equally effective in reducing knee pain over a 26-week period, as no statistically significant difference was observed thereafter.

Another study done by Grecomoro et al. among 40 patients with knee OA divided them into two groups. It was found that the intensity of knee pain decreased much more rapidly in the group treated with combined HA plus dexamethasone than in the group treated with HA alone. Additionally, the combined group experienced lower overall pain levels than the group that received HA alone. Improvement in physical activity, as evidenced by improved joint mobility, was more rapid and better in the patients who received combined injections than those who received only HA. The study, however, did not grade OA, as all respondents were grouped together, making it difficult to determine if clinical improvements were influenced by the severity of knee OA.

Also, Uganath et al. in India demonstrated that the combination of both medications produces a synergistic effect, which is rapid and prolonged in the improvement of knee pain [30]. Their evaluation was done at the first, fourth, and twelfth weeks following administration of the medications. The improvement in pain scores in the combined group compared to the hyaluronic acid group was found to be statistically significant (p<0.05).

We hypothesized that the addition of triamcinolone to hyaluronic acid offers better treatment response in terms of pain relief and physical function than hyaluronic acid only in the treatment of Kellgren and Lawrence grade 2 and 3 knee OA. The aim of this study was to compare the efficacy of combined intra-articular injections of hyaluronic acid and triamcinolone versus hyaluronic acid alone in pain relief of Kellgren and Lawrence grade 2 and 3 knee OA. This was achieved by comparing the pain scores as well as the difference in physical function in both groups of patients.

## Materials and methods

Study design

This was a prospective, randomized, double-blind controlled study that included patients between 40 and 75 years who attended the orthopaedic clinic of the University of Benin Teaching Hospital (UBTH), Benin City, with the diagnosis of knee osteoarthritis. The study was done from May 2023 to April 2024.

Inclusion and exclusion criteria

Participants included consented patients who were in attendance in the UBTH Orthopaedic clinic, aged between 40 and 75 years, with Kellgren and Lawrence grade 2 and 3 knee OA not requiring any surgical intervention. The exclusion criteria included (1) patients with secondary knee osteoarthritis, (2) patients with other lower limb abnormalities, including hip OA, (3) patients with knee joint infection, (4) allergy or hypersensitivity to the study medications, (5) those who have had an intra-articular steroid or hyaluronate injection previously, (6) patients who were immunosuppressed such as cancer, HIV patients, (7) diabetes patients, (8) pregnant women, and (9) patients with Kellgren and Lawrence class 0, 1 and 4 knee OA.

Sampling technique

Patients were recruited into either of the two groups using a simple random technique. Patients who presented with knee OA to the UBTH Orthopaedic clinic who met the inclusion criteria and gave consent were recruited into one of the two groups, Group A (triamcinolone + hyaluronic acid + lidocaine + sterile water), Group B (hyaluronic acid + lidocaine + sterile water), by a simple balloting method.

Sample size determination and allocation

Sample size was determined using the formula for a randomized controlled trial. A sample size of 27 subjects in each group was sufficient to detect a clinically important difference between groups in reducing pain. To compensate for dropouts due to a high rate of defaults in patient follow-up, 18% was added. The sample then increased to 66 respondents, with 33 in each of groups A and B. The total sample size was 66 patients with 33 patients in each group; Group A (patients for intra-articular injection of hyaluronic acid 20 mg (2 ml) + triamcinolone 40 mg (1 ml) + 1% lidocaine (5 ml) + 2 ml of sterile water) and group B (patients for intra-articular injection of hyaluronic acid 20 mg (2 ml) + 1% lidocaine (5 ml) + 3 ml of sterile water). An investigator's proforma was designed to record details of patients’ personal and clinical data.

Patients’ selection

Patients who were in attendance at the UBTH Orthopaedic clinic that met the inclusion criteria and gave consent were clerked by the researcher using the proforma and entered into the study if they had Kellgren and Lawrence 2 and 3 knee OA. Grade 2 = Definite osteophytes, possible narrowing of joint space (Mild), and Grade 3= Moderate diminution of joint space, moderate multiple osteophytes (Moderate). Patients were recruited sequentially as they attended the orthopaedic clinic at UBTH. Once the patient's eligibility was determined, they were assigned to a group using a simple balloting technique supervised by the research assistant. Serial numbers 1 to 66 were written on small pieces of paper and placed in an envelope. Patients who presented to the clinic and met the inclusion criteria were asked to pick a piece of paper from the envelope. Based on the number written on the piece of paper they picked, they were assigned to either group A or B. Patients who picked odd numbers were recruited into Group A and received medications (hyaluronic acid, triamcinolone, lidocaine, and sterile water) for intraarticular injection, while those who picked even numbers were recruited into Group B and received medications for Group B (hyaluronic acid, lidocaine, and sterile water).

Ethical considerations

The study was conducted in total compliance with the Helsinki Declaration, and written informed consent was obtained from all respondents. Approval and RCT registration were also obtained from the University of Benin Teaching Hospital Health Research Ethics Committee (approval number: ADM/E22/A/VOL.VII/148301961).

Technique

For group A, 2 ml (20 mg) of hyaluronic acid, 1 ml (40 mg) triamcinoloneacetonide, 5 ml of 1% plain lidocaine, 2 ml of sterile water were injected intra-articularly into the affected knee, while for group B, the agents intra-articularly injected into the affected knee were 2 ml (20 mg) of hyaluronic acid, 5 ml of 1% plain lidocaine, 3 ml of sterile water. The technique was standardised for both Group A and B as follows: all procedures were performed in the orthopaedic theatre of UBTH following strict aseptic measures. The patient lay supine with the knees extended, and a roll of blanket was placed behind the knee to be injected; this helped to place it into about 10 degrees of flexion. The lower limb was cleaned from the mid-thigh to the mid-leg sequentially using methylated spirit and 10% povidone iodine. Drapes were applied, exposing only the knee. The agents to be injected for either group of patients were withdrawn into a 10 ml syringe by the research assistant, who had the patients’ proforma forms and knew which group each patient belonged to. The edge of the patella was identified, and an entry point about 5 mm posterior to it on the medial aspect of the knee was also located. An 18-G cannula was inserted perpendicular to the long axis of the limb, aimed slightly anterior toward the patella to enter the space between it and the femoral condyles. This was confirmed by aspiration of synovial fluid. The withdrawn agents were then injected into the knee by the principal researcher. The cannula was withdrawn, and a sterile dressing was placed over the injection site. Notably, when knee effusion was present in any respondent in either group, 10 ml of it was first aspirated before the intra-articular injections were administered.

Standard outcome measures

Patients in both groups were evaluated using the 11-point NRS for pain and the short-form WOMAC function scale for physical function status [31,32]. The evaluation was conducted at the following periods: 0 week (prior to the injections) and 2, 4, and 12 weeks after administration of the various agents. Following the evaluation, the scores of each patient were entered into their proforma forms by the research assistant, and at the end of the 12th week post-injection follow-up, these forms were all handed over to the principal researcher by the research assistant, who had kept the records all the while. Both patients and the principal investigator were blinded to the process of allocating the patients into the group, the type of treatment, and the assessment of outcome. The patients’ group was only reviewed at the end of data collection to obviate bias.

Methods of data analysis

Data was collected during a face-to-face interview with the aid of a proforma. This data was coded and entered into an Excel spreadsheet and analyzed using the Statistical Package for the Social Sciences (SPSS) software, version 22 (IBM Corp., Armonk, NY). Categorical variables were expressed as numbers and percentages, and continuous variables were summarized as mean and standard deviation. The Chi-square test was used to compare categorical variables between groups. The student’s t-test was used to compare continuous variables between the two groups. A p-value of < 0.05 was considered statistically significant.

## Results

A total of 66 patients were recruited for this study, with 33 in each group. No patient was lost to follow-up. The sociodemographic characteristics are shown in Table 1.

**Table 1:** Sociodemographic characteristics of the respondents

Variables	Group A	Group B	p-value
	n=33	n=33	
	Freq (%)	Freq (%)	
Age (years)			
Means	54.69±12.49	58.55±9.88	0.17
Sex			
Male	9 (27.3)	13 (39.4)	0.43
Female	24 (72.7)	20 (60.6)	

**Figure 1:**
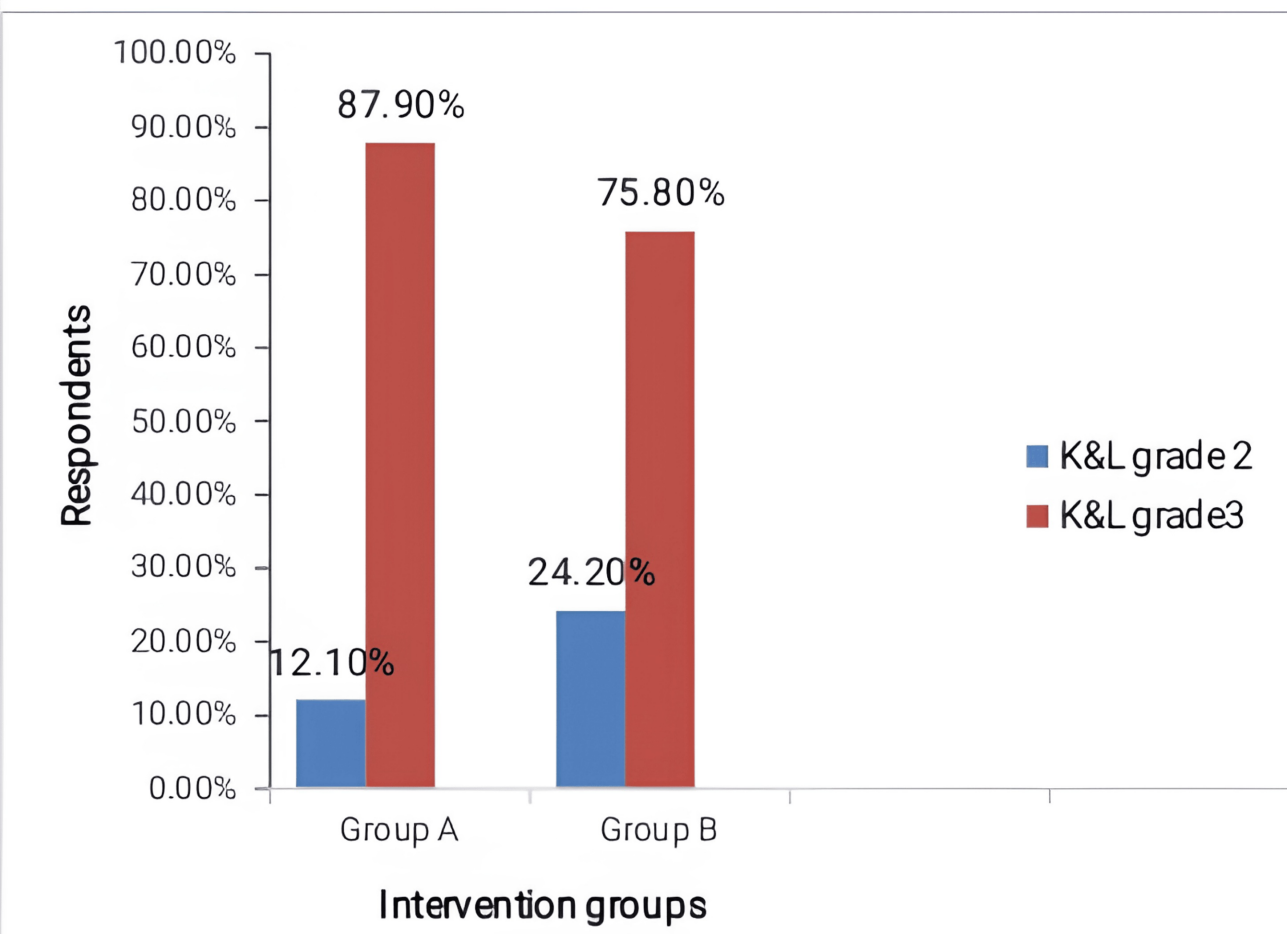
Grouped bar chart showing the severity of Knee OA Group A: Respondents who received combined hyaluronic acid and triamcinolone; Group B: Respondents who received hyaluronic acid.

Table 1 shows that the mean age of both groups was similar. The majority of the respondents were females. The severity of knee OA is represented in Figure 1. The majority of respondents (>70%) in both groups had K & L grade 3 knee OA.

Comparison of pain scores

Table 2 below shows that the reduction in mean pain scores was better and rapid among group A respondents than group B respondents.

**Table 2:** Mean pain scores for respondents in group A and B at 0, 2, 4 and 12 weeks

Week	Group A mean pain scores	Group B Mean pain scores
0	7.58±1.35	8.27±1.31
2	1.73±1.91	4.30±1.31
4	1.52±1.60	2.97±1.21
12	1.27±1.33	2.51±2.27

In Table 3 below, the respondents in Group A consistently had greater changes in their mean pain scores throughout the period of the study compared to respondents in Group B. These improvements in pain scores were all statistically significant (p = 0.001).

**Table 3:** Comparison of the change in pain scores between Groups A and B

Weeks	Group A	Group B	t	df	p	Mean diff.
	n=33	n=33				
0&2weeks	5.85±1.46	3.97±1.21	5.69	64	0.001	1.88
0&4 weeks	6.06±1.09	5.30±1.31	2.56	64	0.001	0.76
0&12week	6.30±1.05	5.76±2.14	3.73	64	0.001	0.54

Comparison of the difference in physical function scores

Table 4 below shows a significant and rapid improvement in the mean physical function scores for Group A respondents compared to those in Group B respondents following commencement of treatment. The improvement in the scores was highest at the 2nd and 12th week, respectively, for both groups A and B.

**Table 4:** The mean WOMAC scores for respondents in groups A and B at 0, 2, 4 and 12 weeks WOMAC: Western Ontario and McMaster Universities Osteoarthritis Index.

Week	Group A	Group B	P
0	12±0.00	12.00±0.00	0.001
2	3.27±2.54	7.27±1.57	
4	3.88±3.08	5.82±2.02	
12	3.39±2.67	5.55±3.13	

The comparison of the improvement in physical function scores is shown in Table 5 below. The respondents in Group A had significant changes (improvement) in their mean WOMAC scores at 2, 4, and 12 weeks compared to the group B respondents (p = 0.001).

**Table 5:** Comparison of the change in WOMAC (physical function) scores in both groups WOMAC: Western Ontario and McMaster Universities Osteoarthritis Index.

Weeks	Group A	Group B	t	df	p	Mean diff.
	n=33	n=33				
0&2	8.73±2.54	4.73±1.57	7.69	64	0.001	4
0&4	8.12±3.08	6.18±2.02	3.02	64	0.001	1.94
0&12	8.61±2.67	6.45±3.13	4.39	64	0.001	2.16

## Discussion

This study found a statistically significant reduction in pain scores and improvement in physical function in patients with K&L 2 and 3 knees OA who received combined intra-articular triamcinolone and HA as compared to those who received HA only (p=0.001). Thus, this study shows that the addition of triamcinolone to HA offers better treatment response in terms of pain relief and physical function in the treatment of K&L 2 and 3 knee OA compared to HA alone (p=0.001).

The mean age of the respondents recruited was 54.69±12.49 years and 58.55±9.88 years in groups A and B, respectively. There was no statistical difference in the age distribution between the two groups. There was a female preponderance in both groups. This indicates a greater prevalence of knee OA among females. A possible reason for this is due to the decline in oestrogen production as women age. This finding is similar to several other studies that showed that knee OA is more prevalent in the female gender [6,11]. More than two-thirds of the respondents had K&L grade 3 knee OA, while the remainder had K&L grade 2 knee OA. The reason for the greater prevalence of grade 3 knee OA compared to grade 2 knee OA is usually due to late or delayed presentations; usually, most respondents would have tried other forms of self-help before presenting to the hospital for proper care.

The mean pain scores for respondents in both groups were high at presentation, as the majority of the respondents had severe knee pain. The mean pain scores reduced significantly following the administration of intra-articular injection of triamcinolone plus HA for group A and HA only for group B. The reductions in mean pain scores for both groups were maintained up to the 12th week post-intra-articular injection. However, the mean change in pain scores was greater and more rapid in patients in group A than in Group B, which was statistically significant (p=0.001). The knee pain in the group A respondents decreased from severe to mild, while that of the group B respondents also decreased from severe to moderate. The implication of this is that combined intra-articular injection of triamcinolone and HA gives better pain relief with resultant lower pain scores than HA alone in the treatment of knee OA. Both HA and triamcinolone have been found to be individually efficacious in relieving pain associated with knee OA, with limitations in their extent of pain relief when used separately [4]. The resultant better pain relief by a combination of the two drug agents suggests a synergistic activity by both drugs.

Uganath et al. in India reported similar findings to this study, demonstrating that the combined medications produce a synergistic effect that is both rapid and prolonged in the improvement of knee pain [30]. Their evaluation was done at the first, fourth, and 12th weeks following administration of the medications, and an improvement in the pain score in the combined group compared to the hyaluronic acid group was found to be statistically significant. Another study done by Ozturk et al. in Turkey found that both groups experienced improvement in pain; however, the HA plus triamcinolone group had superior early pain relief compared to the HA alone group [27]. Their study, in addition, monitored both groups of patients with MRI, which was not done in our study due to the high cost.

Similarly, Petrella et al. in a multicentre study found that combined HA and triamcinolone provides better, early, and rapid pain relief compared to HA only [28]. However, the majority of the respondents in their study were Caucasians. Furthermore, the findings of Grecomoro et al. among 40 patients divided into two groups, with one receiving intra-articular HA plus dexamethasone and the other group receiving HA only, are similar to this study. Both groups had a reduction in knee pain after the first week; however, the intensity of reduction in knee pain was more rapid in the combined group [29]. Also, the combined group had overall lower pain levels compared to their HA-only group, which is similar to this study. However, a major difference was the use of dexamethasone in place of triamcinolone as the steroid.

Another study done by Hangody et al. comparing combined intra-articular hyaluronic acid plus triamcinolone with hyaluronic acid or saline for the treatment of knee OA had similar findings with this study at one and three weeks only [4]. The group that received combined intra-articular hyaluronic acid plus triamcinolone was found to have statistically significant better pain relief than the group that received hyaluronic acid only. However, the improvement in pain was found to be similar in both groups of patients from six weeks, which is different from the findings in this study. This difference may be due to the grade of OA recruited for this study, as the majority of the respondents had mild knee OA, and nearly all the respondents were Caucasians [4].

Moreover, Campos et al., in a study among 104 patients divided into two groups, found that the patients who received combined intra-articular injection of triamcinolone and hyaluronic acid had faster and better pain relief than the patients who received hyaluronic acid only, up until the fourth week post-injection, where there was no difference between the two groups [26]. The findings of similarly reduced pain scores in both groups after the fourth week by Campos et al. differ from this study, which showed continuous superiority of the combined group up to the twelfth week of evaluation; this difference may have been due to the continuous use of analgesics by both groups in the Campos et al. study.

Both groups had similar physical function scores at presentation, and they had severe limitations of their physical function using the WOMAC scale. Following intra-articular administration of triamcinolone and hyaluronic acid to patients in group A and HA alone to patients in group B, the mean WOMAC scores reduced in both groups at two, four, and 12 weeks. The improvement in the mean WOMAC scores was maximum at week 2 for group A (3.27) and at week 12 for group B (5.55), indicating that the group A respondents had better and faster improvement in physical functions than the group B respondents.

The mean difference in the WOMAC physical function scores was greater in group A than in group B, and this difference was maintained even up to the 12th week post-intra-articular injections. This difference was statistically significant (p=0.001), indicating that group A showed better improvement in physical functions compared to group B. Respondents in group A had improvement in their physical function from severe limitations of physical function to mild limitations of physical function following intra-articular administration of triamcinolone and HA, while respondents in group B had improvements in their physical function from severe difficulty to moderate difficulty following administration of HA only, and this improvement was sustained throughout the period of study.

The findings in this study differ from those of the Ozturk et al. study, which found no significant difference in physical function between the groups following the administration of triamcinolone plus HA and HA alone. However, they found improvement in physical function in both groups [27]. The reason for this may be due to the unequal subsample sizes, as the triamcinolone plus HA group had 16 respondents compared to the HA alone group, which had 24 respondents.

A similar finding was reported in this study by Hangody et al., who found better improvement in physical function outcomes in the group that received combined intra-articular hyaluronic acid plus triamcinolone compared to the group that received hyaluronic acid only [4]. However, Campos et al., in a study among 104 respondents with knee OA, divided into two groups of 52, found using the WOMAC scores that the group receiving combined medications had early and superior improvements in physical function compared to the group receiving HA only [26]. They, however, noticed that the improvement in the physical functions became the same in both groups after the fourth week post-injection, which is different from the findings in this study; this difference may have been due to the continuous use of analgesics in both groups of patients in their study.

Based on the findings of this study, which are consistent with those of previous studies outlined above, patients with K&L grade 2 and 3 knee OA should have combined intra-articular injection of triamcinolone and HA as a preferred treatment option rather than hyaluronic acid alone. A multicentre study is recommended to further validate the findings of this study in the West African sub-region. Also, further study on the combination of triamcinolone and HA over an extended period of one year is encouraged in order to evaluate if this type of treatment can be given once yearly to sufferers of knee OA. However, this study was limited by the short duration of follow-up of the patients. This was for 12 weeks post-intra-articular injection.

## Conclusions

Combined intra-articular injection of triamcinolone and HA gives a faster, better and sustained reduction in pain scores in patients with K&L grade 2 and 3 knee OA than intra-articular HA only. The combined drugs also improve physical function in patients with K&L grade 2 and 3 knee OA more rapidly and effectively compared to intra-articular HA alone. Thus the combination of Triamcinolone and HA appears to give a better synergistic effect in the relief of pain and improved physical function in patients with K&L grade 2 and 3 knee OA than intra-articular HA only.
